# Clinical Evaluation of Heart Failure: Agreement among Tests

**DOI:** 10.1371/journal.pone.0161536

**Published:** 2016-08-18

**Authors:** Amit K. Pandey, William F. Penny, Valmik Bhargava, N. Chin Lai, Ronghui Xu, H. Kirk Hammond

**Affiliations:** 1 Department of Medicine, University of California San Diego, San Diego, CA, United States of America; 2 Department of Medicine, VA San Diego Healthcare System, San Diego, CA, United States of America; 3 Department of Family Medicine and Public Health, University of California San Diego, San Diego, CA, United States of America; 4 Department of Mathematics, University of California San Diego, San Diego, CA, United States of America; Merck & Co., UNITED STATES

## Abstract

Methods commonly used clinically to assess cardiac function in patients with heart failure include ejection fraction (EF), exercise treadmill testing (ETT), and symptom evaluation. Although these approaches are useful in evaluating patients with heart failure, there are at times substantial mismatches between individual assessments. For example, ETT results are often discordant with EF, and patients with minimal symptoms sometimes have surprisingly low EFs. To better define the relationship of these methods of assessment, we studied 56 patients with heart failure with reduced EF (HFrEF) who underwent measurement of ETT duration, EF by echocardiography, quantitative symptom evaluation, and LV peak dP/dt (rate of left ventricular pressure development and decline, measured invasively). Correlations were determined among these four tests in order to assess the relationship of EF, ETT, and symptoms against LV peak dP/dt. In addition, we sought to determine whether EF, ETT, and symptoms correlated with each other. Overall, correlations were poor. Only 15 of 63 total correlations (24%) were significant (p < 0.05). EF correlated most closely with LV peak -dP/dt. Linear regression analysis indicated that EF, ETT, and symptoms taken together predicted LV peak dP/dt better than any one measure alone. We conclude that clinical tests used to assess LV function in patients with HFrEF may not be as accurate or correlate as well as expected. All three clinical measures considered together may be the best representation of cardiac function in HFrEF patients currently available.

## Introduction

Heart failure (HF) is the most common diagnosis for nonelective hospitalization in individuals over the age of 65 in the US [1] and is increasing in prevalence. There are several methods commonly used to assess clinical status of patients with HF, including ejection fraction (EF), exercise treadmill testing (ETT), and symptom assessment. The focus of the current study is to evaluate how well these tests relate to one another and how accurately they predict left ventricular (LV) function assessed by a less load-dependent measure of contractile function than ejection fraction, namely LV peak dP/dt (the maximal rate of LV pressure development and decline, measured invasively).

Despite the widespread use of EF, ETT, and symptom assessment, there is a scarcity of publications examining correlations between these parameters. The most studied relationship is that between exercise capacity and EF. Subjects with HF have reduced exercise capacity compared to patients with normal LV function [2–6], which is consistent with clinical intuition. However, among patients with HF with reduced EF (HFrEF), exercise capacity is variable and does not correlate with EF [7,8]. Furthermore, studies indicate that β-adrenergic receptor antagonists and angiotensin converting enzyme inhibitors do not predictably increase exercise performance in subjects with HF [9,10], although both improve LV function and survival. Symptoms have also been shown to correlate poorly with hemodynamic, echocardiographic, and laboratory data [11]. Similarly, clinical classification of HF based on symptoms using the New York Heart Association criteria does not correlate with exercise duration [12]. The lack of correlation among markers of LV function has previously been incompletely described. In the current study we quantify these relationships and compare these tests to LV peak dP/dt, a less load-dependent measure of LV function than EF.

The present study examines the correlations among four important measures used to assess LV function in patients with HF: ETT, EF, symptoms, and LV peak dP/dt. Of the measures of LV contractile function available, the rate of LV pressure development (LV peak +dP/dt) is the most precise [12,13]. Compared to EF, LV peak +dP/dt is less dependent on preload and afterload, and more accurately reflects intrinsic LV contractile function [14,15,16]. However, measuring LV peak dP/dt requires placement of a pressure transducer in the LV cavity, and therefore is rarely used clinically. A strength of the current study is that LV peak dP/dt was measured as a prerequisite in a clinical study of 56 subjects enrolled in an NIH-funded HF trial [17]. It was measured before and during dobutamine infusion, providing a little-used but accurate assessment of LV contractility against which the other less invasive measures of LV function could be compared. Using data acquired from 56 subjects with symptomatic HFrEF (before gene transfer), we had two goals: 1) compare LV peak dP/dt to other commonly used surrogate measures of LV function; and 2) determine the correlation among commonly used clinical measures of LV function.

## Materials and Methods

### Study Patients

Data analyzed in the present study were baseline values collected from subjects with symptomatic HFrEF in a recently reported clinical trial [17]. Here we present data collected prior to randomization, which do not reflect effects of the test article. The focus was exclusively on the correlation of pre-randomization tests used to evaluate LV function (previously unpublished data). Data were gathered from 56 participants who underwent a series of clinical tests as part of the trial. Patients with symptomatic but stable HFrEF were enrolled in the clinical trial. The key inclusion criteria were: 18–80 years of age; symptomatic HF; EF ≤ 40%; implanted cardiac defibrillator; at least one coronary artery (or conduit) >50% patent. The key exclusion criteria were: decompensated HF; severe symptomatic coronary disease; liver disease; and pregnancy. Details of the trial were previously published [17].

The study was approved by the NIH Recombinant DNA Advisory Committee (RAC) and the NIH Data and Safety Monitoring Board. The research involving human subjects was approved by the Institutional Review Boards at each of the participating sites, and adhered to the principles expressed in the Declaration of Helsinki. The majority of patients in the study were enrolled at Veterans Affairs San Diego Healthcare System, and were approved by IRB H130167. Written informed consent was obtained from all participants, and the informed consent forms were approved by all of the IRBs. The selection of subjects for the study adhered to NIH guidelines. No “rules of human categorization” were required by the NIH. The NIH requested targets for enrollment of female participants and minority populations, as defined by the NIH.

### Data

Pre-randomization data were analyzed from the following clinical tests:

1. **ETT Duration:** Each subject performed two qualifying ETTs. A modification of the modified Naughton protocol was used to measure exercise duration.
2. **EF:** Obtained from echocardiography, measured before and during dobutamine infusion (5, 10, and 20 μg/kg/min).
3. **LV peak dP/dt:** The peak rates of LV pressure development (+dP/dt) and decline (-dP/dt) were obtained during cardiac catheterization, before and during dobutamine infusion (5, 10, and 20 μg/kg/min).
4. **Symptoms:** The Kansas City Cardiomyopathy Questionnaire (KCCQ) was used. A numerical score was calculated based on patient responses to the questionnaire.

### Exercise Treadmill Testing

A modification of the modified Naughton protocol was used for exercise testing. The primary endpoint was total time that the subject performed exercise (symptom-limited). Treadmill speed was 1.5 mph for stage 1; all subsequent stages had a speed of 2.5 mph. Stage duration was 1 min for stages 1 and 2; all other stages lasted 2 min. Grade was 0% for stages 1 and 2; grade increased by 2.5% every 2 min starting at the end of stage 2. Heart rate, blood pressure, and ECG were recorded during each stage and patient symptoms were monitored. Subjects were asked to discontinue β-adrenergic receptor antagonists (β-blockers) for one day prior to testing.

### Echocardiography

LV EF was assessed by conventional 2D echocardiography before and during graded intravenous dobutamine infusion. End-diastolic and end-systolic volumes were calculated using the method of discs (Simpson’s Rule). This method summates volumes of multiple cylinders of equal height along the LV that are obtained from the apical 4-chamber and 2-chamber views. Ejection fraction was then calculated as: EF = [(EDV-ESV)/EDV] x 100. Definity^®^ or Optison^®^ (contrast agent) was used to optimize identification of endocardial borders. Three doses of dobutamine were administered (5, 10, and 20 μg/kg/min, 5 min each). EF was measured before and during each dose of dobutamine.

### LV Contractile Function Assessment

A 5F JR4 fluid-filled guide catheter was placed retrograde into the LV cavity and a Millar micromanometer catheter was advanced through the lumen of the guide catheter. Pressure data were acquired at 500 Hz and the measurements were recorded simultaneously with both the Millar and fluid-filled pressure transducers. Data from the fluid-filled pressure transducer were used to calibrate the Millar transducer pressure [18]. Data were acquired at baseline and during graded dobutamine infusion (5, 10, and 20 μg/kg/min, 5 min each), and were analyzed during the last 30 s of each 5 min infusion for LV peak +dP/dt and LV peak -dP/dt. Premature beats and the subsequent two beats were excluded, and dP/dt values for all remaining beats in the 30 s interval were averaged.

### Symptom Assessment

We used the KCCQ [19], but excluded two questions pertaining to the participant’s knowledge of HF; this was done before any correlations were performed. The 21 questions scored 0 to 4 yielded total symptom scores ranging from a minimum of 0 to a maximum of 84, with lower scores denoting more favorable states (less symptoms) and higher scores denoting less favorable states (more symptoms).

### Data Analysis

Statistical analysis was performed using both *R* and *GraphPad Prism 5* software. Correlations were performed among the four tests of LV function: ETT, EF, LV peak dP/dt (both +dP/dt and -dP/dt), and symptoms. Pairwise Pearson’s correlations were calculated among all endpoints of these four tests: EF, stimulated EF, ETT duration, symptom score, LV peak +dP/dt, stimulated LV peak +dP/dt, LV peak -dP/dt, and stimulated LV peak -dP/dt. “Stimulated” indicates measurements made during dobutamine infusion, and represents the maximal value recorded during any of the three graded doses. These correlations yielded r-values, which were then tested for significance from zero and the corresponding p-values reported. These p-values were not adjusted for multiple comparisons, as they were seen as mostly descriptive of the strength of the correlation, and the analysis itself was exploratory. Linear regression analysis was performed to determine whether clinical measures of LV function (EF, ETT duration, symptom score) predicted LV peak dP/dt. Each clinical test was considered an independent variable (x) and each endpoint of LV peak dP/dt was considered a dependent variable (y). Multiple linear regression analysis was also performed with the three clinical markers as the multiple independent variable and each endpoint of LV peak dP/dt as the dependent variable. Comparison of R^2^ values between these linear regression analyses helped determine whether the three clinical measures taken together predicted LV peak dP/dt better than each alone. Of note, r-values represented correlation coefficients yielded by Pearson’s correlations, while R^2^ values represented coefficients of determination yielded by linear regressions.

## Results

### General Characteristics

Table 1 lists the general clinical characteristics of the study population, including historical variables and medications. Of the study patients, 48% had ischemic and 52% had non-ischemic etiology of heart failure. The subjects predominantly had New York Heart Association Class 2 or 3 HF. A prior MI had occurred in 56% and 40% had undergone coronary artery bypass grafting. Table 2 lists the mean values of key measures of LV function yielded by the four tests considered in this study.

**Table 1:** Characteristics of Study Population (n = 56).

History	Male (%)	91
	Age (year)	63 ± 9
	Weight (kg)	95 ± 24
	NYHA Class (%)	
	• Class 1	0
	• Class 2	45
	• Class 3	51
	• Class 4	4
	Ischemic Etiology (%)	48
	Non-ischemic Etiology (%)	52
	Prior MI (%)	56
	Coronary Artery Bypass Surgery (%)	40
	Percutaneous Coronary Intervention (%)	40
	History of Hypertension (%)	71
	History of Diabetes (%)	47
Medications	Beta Blockers (%)	95
	Digoxin (%)	36
	ACE Inhibitor/ARB (%)	88
	Diuretic (%)	73
	Spironolactone/Eplerenone (%)	38

Values represent mean ± standard deviation. NYHA, New York Heart Association; MI, Myocardial Infarction; ACE, Angiotensin Converting Enzyme; ARB, Angiotensin Receptor Blocker.

**Table 2:** Mean Values of Key Measures of LV Function (n = 56).

Measure of LV Function	Mean ± SD
EF (%)	30 ± 9
ETT Duration (min)	7.2 ± 3.3
Symptom Score	33.1 ± 16.3
LV peak +dP/dt (mmHg/s)	984 ± 233
LV peak -dP/dt (mmHg/s)	-1093 ± 269

LV, Left Ventricular; SD, Standard Deviation; EF, Ejection Fraction; ETT, Exercise Treadmill Test.

### Pearson’s Correlations

Overall, correlations among markers of LV function were poor. Although general trends can be seen, correlations were significant in a minority of comparisons. In total only 15 of 63 correlations (24%) were significant. The mean of the absolute values of all correlation coefficients (r) was 0.26, with a range from < 0.01 to 0.56. Table 3 lists correlation coefficients and p-values for each pairwise correlation between measures of LV function for the entire study population. A subgroup analysis was also performed to determine whether correlations varied when the population was split by etiology of HF (ischemic vs. non-ischemic). This analysis yielded 42 additional correlations (21 in each etiology subgroup). Correlations did not appreciably vary as compared to the entire population, and thus the data are not presented.

**Table 3:** Correlations Among Markers of LV Function.

	ETT Duration	Symptom Score	LV Peak +dP/dt	Stimulated LV Peak +dP/dt	LV Peak-dP/dt	Stimulated LV Peak -dP/dt
EF	r = 0.13	r = -0.04	**r = 0.40**	r = 0.25	**r = -0.51**	**r = 0.28**
	(p = 0.390)	(p = 0.760)	**(p = 0.004)**	(p = 0.080)	**(p < 0.001)**	**(p = 0.047)**
Stimulated EF	r = 0.23	r = -0.12	r = 0.22	r = 0.15	**r = 0.42**	r = 0.24
	(p = 0.119)	(p = 0.387)	(p = 0.130)	(p = 0.311)	**(p = 0.002)**	(p = 0.093)
ETT Duration	-	**r = -0.41**	r = 0.03	r = 0.11	r = 0.28	r = 0.22
		**(p = 0.004)**	(p = 0.823)	(p = 0.480)	(p = 0.058)	(p = 0.156)
Symptom Score	-	-	r = -0.21	**r = -0.37**	r = -0.24	**r = -0.38**
			(p = 0.135)	**(p = 0.007)**	(p = 0.085)	**(p = 0.006)**

r represents correlation coefficient from Pearson’s correlation. EF, Ejection Fraction; ETT, Exercise Treadmill Test; LV, Left Ventricular.

**Fig 1:**
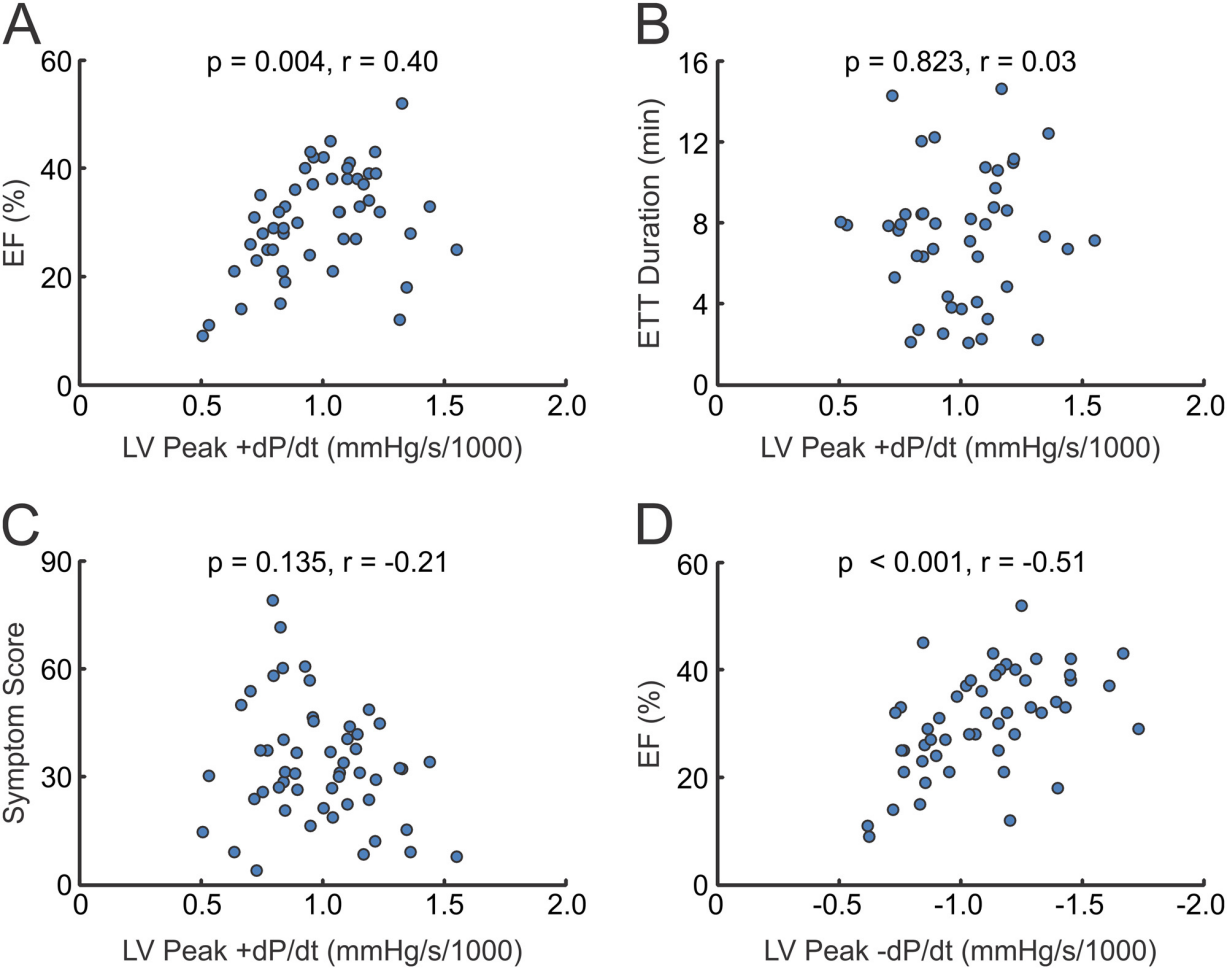
Selected Graphs of Correlations Between Tests of LV Function. Graphs represent Pearson’s correlations, with p-values and correlation coefficients (r) listed. EF, Ejection Fraction; ETT, Exercise Treadmill Test; LV, Left Ventricular.

In the analysis of the entire population, 7 out of 21 correlations (33%) were significant (Table 3**)**. The mean of the absolute values of r was 0.25, with a range from 0.03 to 0.51. EF correlated with LV peak +dP/dt (r = 0.40, p = 0.004), LV peak -dP/dt (r = -0.51, p < 0.001) and stimulated LV peak -dP/dt (r = 0.28, p = 0.047). Stimulated EF correlated with only LV peak -dP/dt (r = 0.42, p = 0.002). Symptom score correlated with ETT (r = -0.41, p = 0.004), stimulated LV peak +dP/dt (r = -0.37, p = 0.007), and stimulated LV peak -dP/dt (r = -0.38, p = 0.006). Of the significant correlations in this group, 6 out of 7 were between LV peak dP/dt and one of the common clinical measures. Notably, EF and stimulated EF each showed no correlation with ETT or symptom score, and ETT did not correlate with LV peak +dP/dt. Fig 1 depicts scatter plots of data for the Pearson’s correlations between LV peak +dP/dt and each of the clinical tests (EF, ETT, symptoms), as well as LV peak -dP/dt and EF, which yielded the highest r value.

### Linear Regressions

Table 4 lists coefficients of determination (R^2^) and p-values for linear regressions among the tests of LV function. All linear regression models were weak, with R^2^ values less than 0.3. However, multiple linear regression yielded a substantially higher R^2^ value in each case of LV peak dP/dt (LV peak +dP/dt, stimulated LV peak +dP/dt, LV peak -dP/dt, stimulated LV peak -dP/dt), indicating that the clinical markers taken together predict LV peak dP/dt better than any single marker does.

**Table 4:** Linear Regressions Among Markers of Heart Function.

Independent Variable	Dependent Variable			
	LV Peak	Stimulated LV	LV Peak	Stimulated LV
	+dP/dt	Peak +dP/dt	-dP/dt	Peak -dP/dt
EF	R^2^ = 0.09	R^2^ = 0.04	**R**^**2**^ **= 0.23**	R^2^ = 0.07
	(p = 0.055)	(p = 0.223)	**(p < 0.001)**	(p = 0.091)
Symptom Score	R^2^ = 0.05	**R**^**2**^ **= 0.12**	R^2^ = 0.05	**R**^**2**^ **= 0.10**
	(p = 0.137)	**(p = 0.024)**	(p = 0.130)	**(p = 0.036)**
ETT Duration	R^2^ < 0.01	R^2^ < 0.01	R^2^ = 0.08	R^2^ = 0.04
	(p = 0.880)	(p = 0.634)	(p = 0.064)	(p = 0.173)
EF, Symptom Score, ETT Duration	R^2^ = 0.15	R^2^ = 0.16	**R**^**2**^ **= 0.30**	R^2^ = 0.17
	(p = 0.084)	(p = 0.067)	**(p = 0.003)**	(p = 0.063)

R^2^ represents coefficient of determination from linear regression. EF, Ejection Fraction; ETT, Exercise Treadmill Test; LV, Left Ventricular.

## Discussion

The most important finding of the present study is that the clinical measures of LV function commonly used to assess patients with HFrEF (EF, exercise tolerance, symptoms) are not tightly correlated with LV peak +dP/dt, the best estimate of LV contractile function available clinically. By combining the three surrogate measures of LV function (EF, ETT duration, symptom score), one attains a tighter correlation with LV peak dP/dt and stimulated LV peak dP/dt (both positive and negative); however the coefficients of determination, while improved, are weak (R^2^ values ranging from 0.15–0.30; Table 4). Although no single clinical measure was tightly correlated with LV peak +dP/dt, EF showed the highest correlation and ETT duration the lowest. Our finding that LV peak +dP/dt correlated moderately weakly with both EF and symptoms and not at all with ETT duration was somewhat surprising. Overall this study indicates that surrogate measures of LV function in patients with HFrEF are not tightly correlated with LV peak +dP/dt, used as a relative gold standard of LV contractile function. Several possible reasons exist which may explain the lack of correlation among these tests, and are discussed below.

LV peak +dP/dt in healthy subjects and subjects with HFrEF are rare, but our findings were similar to those reported previously, and were 26% lower than normal values [20,21]. For example, combining data previously published yielded the following mean values for LV peak +dP/dt: 1) Normal subjects (n = 10): LV peak +dP/dt = 1337 ± 174 mmHg/s; 2) Subjects with HFrEF (n = 16): LV peak +dP/dt = 872 ± 253 mmHg/s [20,21]. In the present study, subjects with HFrEF (n = 50) had a mean LV peak +dP/dt of 991± 222 mmHg/s, and a range from 636 mmHg/s to 1550 mmHg/s. Our data were consistent with previously published values of LV peak +dP/dt in HFrEF, and were lower than published values for normal subjects. Furthermore, there was a similarly broad range of EFs (9% — 40%), ETT durations (124 s– 854 s), and symptom scores (7–50), suggesting that the spread of data among all four markers of LV function was consistent.

### LV EF vs LV peak dP/dt

Although we found that LV EF was significantly correlated with LV peak +dP/dt (p = 0.004) and LV peak -dP/dt (p < 0.001), the correlation coefficients indicated modest correlations (r = 0.40 and r = -0.51, respectively; Table 3 and Fig 1). A larger sample size may have improved this correlation. We could find no studies that have measured LV EF and LV peak dP/dt in subjects with HFrEF. Variability in EF in such subjects is common from month-to-month and may reflect the load-dependence of ejection phase indices of LV function. Because LV peak +dP/dt occurs prior to aortic valve opening, it is not as susceptible to variations in arterial and aortic impedance and generally is thought to be a better estimate of LV contractile function than EF [14].

Though EF is widely accepted as a surrogate of LV function, EF determination by echocardiography is operator-dependent and affected by the skill of the sonographer performing it. EF also depends on geometric assumptions to calculate volumes from 2D images. In addition, the quality of ultrasound images is often suboptimal, contributing to additional error. In the present study, a single, experienced sonographer made the measurements of LV volumes using contrast agents to enhance endocardial border detection.

In contrast, the measurement of LV pressure only requires proper positioning across the aortic valve, and proper calibration of the LV pressure signal. LV pressure signals were calibrated by a physiologist skilled in the measurement of LV peak dP/dt. Although we are not proposing that patients undergo measurement of LV peak dP/dt, which requires left heart catheterization, we are suggesting that LV EF has limitations as a measurement of LV contractile function.

### ETT Duration vs LV peak dP/dt

There was no correlation between ETT duration and any measure of LV peak dP/dt. ETT duration correlated only with symptom score (r = -0.41, p = 0.004; Table 3). Although previous studies have shown that EF and ETT duration were poorly correlated [7,8], we anticipated that a more direct measure of LV contractile function (LV peak +dP/dt) would reveal a correlation with ETT duration. Nevertheless, LV peak +dP/dt correlated poorly with ETT duration. Based on our findings and these previous papers, ETT duration poorly predicts LV function. The reason may reside in the multiple non-cardiac limitations of exercise duration: respiratory disease, peripheral vascular disease, and variations in skeletal muscle conditioning. Although exercise duration is not commonly used in routine clinical management of patients with HFrEF, it has been used in clinical trials of HF.

### Symptom Score vs LV peak dP/dt

Symptom score was correlated, albeit weakly, with stimulated LV peak dP/dt. Both stimulated LV peak +dP/dt (r = -0.37, p = 0.007) and stimulated LV peak -dP/dt (r = -0.38, p = 0.006) showed modest correlations that were not seen in LV peak +dP/dt or -dP/dt (Table 3). Very few subjects had symptoms at rest, but all were symptomatic with mild to moderate activity. Therefore, a measure of cardiac contractile reserve (stimulated LV peak +dP/dt) would be expected to correlate better with exertional symptoms. Symptom score was better correlated with stimulated LV peak dP/dt than was ETT duration or EF. This correlation with LV peak dP/dt is interesting because of the three clinical measures, symptoms are the least objective. Psychological factors contribute to the perception of symptoms, which indicates that each patient’s answers to the KCCQ cannot be expected to be completely uniform. Despite this limitation, symptom score is the only test in this study that correlated with ETT duration (r = -0.41, p = 0.004; Table 3).

### LV peak -dP/dt

An unanticipated finding was that stimulated LV peak -dP/dt correlated with symptom score. LV peak -dP/dt is the maximal rate of LV pressure decline during diastole, a major determinant of diastolic function. Abnormal diastolic function is associated with increased filling pressures and dyspnea presumably due to altered lung compliance and ultimately pulmonary edema. Therefore it is not surprising that a measure of diastolic function would be linked with symptoms, as so many questions on the KCCQ relate to feelings of shortness of breath.

### Combined Model

Overall, the clinical tests of LV function were poor predictors of LV peak dP/dt. However, when combining the three clinical tests, the resultant R^2^ with each of the four elements derived from LV peak dP/dt (LV peak +dP/dt and -dP/dt, stimulated LV peak +dP/dt and -dP/dt) were higher than any single measurement (Table 4). This analysis suggests that the most effective way to evaluate LV function clinically is to take into account EF, exercise capacity and symptoms, rather than any single marker alone.

### Study Limitations

All subjects considered in this study had EF ≤ 40% (HFrEF). Thus, conclusions regarding correlations among LV peak dP/dt and the common clinical measures in patients with HF with preserved EF (HFpEF) cannot be made based on the present study. It is possible that correlations among markers of LV function would be altered if subjects with HFpEF were included. Future studies will be necessary to elucidate the nature of these relationships.

LV dP/dt is not a commonly used clinical measure, and we are not advocating for a resurrection of invasive monitoring of dP/dt in contemporary heart failure management. Rather, we conclude that the commonly used measures of LV function (EF, exercise capacity, and symptoms) correlate poorly with one another and with LV dP/dt, a time-honored assessment of LV contractile function.

### Clinical Implications

The conclusion that EF, ETT duration, and symptoms correlate modestly or not at all with a direct physiological assessment of LV function (LV peak dP/dt) is interesting. However, the measurement of LV peak dP/dt is invasive and associated with risk. Several studies have described non-invasive estimates of LV peak dP/dt, including methods based on diastolic blood pressure and isovolumic contraction [22], a transthoracic sensor measuring changes in cardiac force [23], and the velocity of the mitral regurgitant jet measured by Doppler echocardiography [24,25]. Though none of these methods replace direct measurement of LV pressure and derivation of LV peak dP/dt, they are promising, safe alternatives that may have a place in the assessment of patients with HFrEF.

A significant conclusion of the present study is that EF, exercise capacity, and symptoms do not correlate well with each other. It is important to note that although these measures correlate poorly and perhaps should not be used independently as single measures of LV function, each has an important clinical role in managing individual subjects. For example, very low EFs are generally associated with reduced longevity. In addition, EF can serve as a marker for whether a patient is responding to therapy. Symptoms will always play a role in patient assessment, even if they are at times marginalized as soft indicators in clinical trials because of their subjectivity. Exercise tolerance might serve as a rough guide to progressive decline in heart function. The conclusion here is not that EF, exercise capacity, and symptoms have no role in clinical assessment of HF, but rather that despite wide belief otherwise, these three measures of extent of HF do not correlate well with each other or with a measure of left ventricular contractile function (LV dP/dt).

### Conclusions

Our findings indicate that overall clinical tests used to assess HFrEF patients correlate poorly with one another and with LV peak dP/dt. Of the three clinical tests considered, EF shows the highest correlation with LV peak dP/dt. EF, ETT, and symptoms may be less effective measures of LV function than commonly believed. Under the limitations of current clinical practice, the most effective means of assessing LV function is to consider EF, exercise capacity, and symptoms together rather than any single measure alone. An awareness of the poor correlation among common clinical tests and LV function may be useful in the assessment of HFrEF patients. Development of non-invasive measures of LV peak dP/dt may be important for future clinical practice.

## Supporting Information

S1 File Data Set. (XLSX)
